# Hereditäre pulmonal-arterielle Hypertonie

**DOI:** 10.1007/s00108-024-01718-y

**Published:** 2024-05-21

**Authors:** Christina A. Eichstaedt, Memoona Shaukat, Ekkehard Grünig

**Affiliations:** 1 Zentrum für Pulmonale Hypertonie, Thoraxklinik am Universitätsklinikum Heidelberg, Röntgenstraße 1, 69126 Heidelberg, Deutschland; 2 Labor für molekulargenetische Diagnostik, Institut für Humangenetik, Universität Heidelberg, Heidelberg, Deutschland

**Keywords:** Lungenhochdruck, Genetische Testung, „Bone morphogenetic protein receptor 2“ (BMPR2), Reduzierte Penetranz, Pulmonale venookklusive Erkrankung, Sotatercept, Pulmonary hypertension, Genetic testing, Bone morphogenetic protein receptor 2 (BMPR2), Reduced penetrance, Pulmonary veno-occlusive disease, Sotatercept

## Abstract

Die hereditäre pulmonal-arterielle Hypertonie (PAH) kann durch Mutation in mindestens 18 Genen ausgelöst werden. Das am häufigsten veränderte Gen ist das für den „bone morphogenetic protein receptor 2“ (*BMPR2*). Auch weitere Gene im selben Signalweg sind als PAH-Gene bekannt. Eine positive genetische Testung ist hilfreich, um Differenzialdiagnosen wie eine pulmonale venookklusive Erkrankung zu sichern, und ermöglicht die Testung gesunder Familienangehöriger. Neben dem Patient*innenkollektiv, dem eine genetische Testung besonders dient, geht dieser Beitrag auf die Art der Vererbung der hereditären PAH ein und gibt einen Einblick in erste Therapien, die den BMPR2-Signalweg wieder ins Gleichgewicht bringen.

## Abstract (en)

Heritable pulmonary arterial hypertension (PAH) can be triggered by at least 18 genes. The most frequently altered gene is the bone morphogenetic protein receptor 2 (*BMPR2*). Further genes from the same pathway are also well known PAH-causing genes. Genetic testing can aid to confirm differential diagnoses such as a pulmonary veno-occlusive disease. It also enables the testing of healthy family members. In addition to the PAH patient population particularly served by genetic testing, this article touches on the mode of inheritance and provides insights into the first treatments soon on the market that rebalance the BMPR2 signaling pathway.

Die pulmonal-arterielle Hypertonie (PAH) ist eine seltene Erkrankung. Eine Erhöhung des pulmonal-arteriellen Widerstands (> 2 Wood-Einheiten) und des mittleren pulmonal-arteriellen Drucks (> 20 mmHg) führen im Verlauf bis zu einer Hypertrophie und Insuffizienz des rechten Herzens [11]. Unspezifische Symptome wie Dyspnoe unter Belastung, Palpitationen und Ödeme sind erste Anzeichen, die bis zu einer Atemnot in Ruhe und Synkopen führen [11]. Eine PAH kann sporadisch als idiopathische PAH (IPAH) auftreten, familiär gehäuft bzw. genetisch bedingt sein (hereditäre PAH [HPAH]) oder durch Umwelteinflüsse wie Medikamente und Toxine bzw. angeborene Herzfehler oder rheumatische Erkrankungen verursacht werden. Von einer HPAH ist die Rede, wenn eine genetische Ursache für die Erkrankung identifiziert wurde oder wenn mindestens zwei Familienmitglieder daran erkrankt sind.

## Genetische Ursachen

Nachdem bei mehreren großen HPAH-Familien im Jahr 2000 zeitgleich das Hauptgen für HPAH entdeckt wurde, das Gen für den „bone morphogenetic protein receptor 2“ (*BMPR2*; [1, 9, 13]), wurden kontinuierlich weitere PAH-auslösende Gene identifiziert. So konnten wir beispielsweise 2017 den Transkriptionsfaktor *KLF2* bei einer HPAH-Familie als krankheitsursächlich und somit als neues PAH-Gen beschreiben [5]. Ein weltweites Expertengremium charakterisierte im Jahr 2023 zwölf Gene als definitiv PAH-assoziiert, drei als moderat assoziiert und weitere als limitiert assoziiert [16]. Dafür wurde eine etablierte Krankheits-Gen-Evaluierungsmatrix der Vereinigung Clinical Genome Resource (ClinGen) eingesetzt, die unter anderem publizierte Fälle, den Expressionsort der Gene und den mechanistischen Zusammenhang mit PAH berücksichtigte und bewertete. Zu den zwölf Genen mit der höchsten Evidenz gehören neben *BMPR2* allein fünf weitere Gene aus dem BMPR2-Signalweg.

## Genetische Diagnostik

Patient*innen, die an einer IPAH, HPAH oder medikamenten- bzw. toxininduzierten PAH erkrankt sind oder eine PAH bei assoziiertem Herzfehler haben, wird eine genetische Diagnostik zur weiteren Abklärung empfohlen [2]. Auch Kindern mit einer PAH und Patient*innen, bei denen der Verdacht auf eine pulmonale venookklusive Erkrankung („pulmonary veno-occlusive disease“ [PVOD]) oder einen Morbus Osler bei PAH besteht, wird eine genetische Diagnostik angeraten [2]. Eine genetische Beratung bereits erkrankter Patient*innen dürfen behandelnde Ärzt*innen durchführen und im Anschluss eine Anforderung zur genetischen Testung stellen.

Ein wichtiger Beitrag der genetischen Diagnostik ist die Bestätigung oder der Ausschluss von Differenzialdiagnosen. PAH-Patient*innen, die in den Genen der Korezeptoren des BMP-Rezeptors 2, ALK1 oder Endoglin, eine pathogene Variante (Mutation) tragen, werden im Laufe ihres Lebens einen Morbus Osler (hereditäre hämorrhagische Teleangiektasie) entwickeln, der durch Teleangiektasien, Epistaxis sowie arteriovenöse Malformationen in der Lunge, aber auch in Gastrointestinaltrakt, Leber und Gehirn gekennzeichnet sein kann [7]. Die Differenzialdiagnose einer PVOD steht hingegen bei Patient*innen im Raum, die eine erniedrigte Diffusionskapazität der Lunge für Kohlenstoffmonoxid (DLCO) haben, Auffälligkeiten der Lunge in der Computertomographie wie verdickte interlobuläre Septen, zentrilobuläre Milchglastrübungen und eine Lymphadenopathie zeigen [11] und/oder schlecht auf eine PAH-spezifische Medikation ansprechen. Genetisch kann der Verdacht auf eine PVOD durch Mutationen im Gen *EIF2AK4* bestätigt werden. Eine gesicherte PVOD-Diagnose kann zu einer schnelleren Evaluation und Listung für eine Lungentransplantation beitragen. Aber auch das Vorhandensein von *BMPR2*-Mutationen reduziert im Schnitt die Überlebenszeit der Patient*innen und geht mit einer schlechteren Hämodynamik einher [6].

Eine genetische Diagnostik sollte alle bekannten PAH-Gene beinhalten

**Figure 1:**
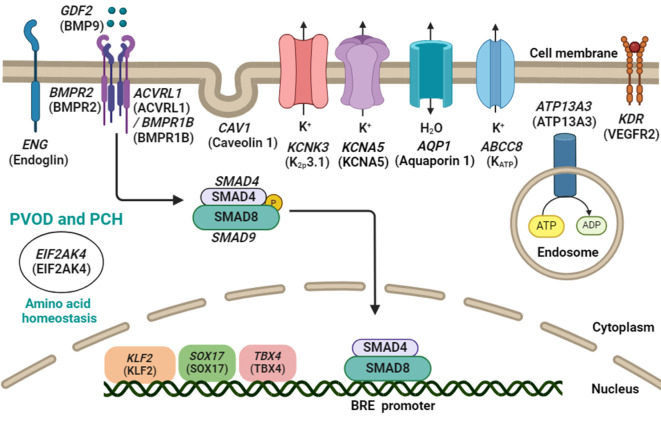

Eine genetische Diagnostik sollte sich gezielt auf die bekannten PAH-Gene konzentrieren. Methode der Wahl ist daher eine Genpaneldiagnostik, die eine hohe Sequenzabdeckung garantiert und eine gezielte Suche nach krankheitsrelevanten Varianten ermöglicht. Am Universitätsklinikum Heidelberg extrahieren wir daher DNA aus einer kleinen Ethylendiamintetraacteat(EDTA)-Blutprobe und wenden unser patentiertes PAH-Genpanel mit allen 18 bekannten PAH-Genen an (Abb. 1). Interpretation und Befundung der identifizierten Varianten erfolgen nach den Richtlinien des American College of Medical Genetics and Genomics [14].

## Vererbung der pulmonal-arteriellen Hypertonie

Die HPAH ist in der Regel eine autosomal-dominant vererbte Erkrankung mit einer reduzierten Penetranz. Das bedeutet, dass die Vererbung einer Mutation, wie etwa einer Mutation im Gen *BMPR2*, von einem Elternpaar ausreicht, um möglicherweise zur Erkrankung zu führen. Interessanterweise bedingt eine genetische Veranlagung für PAH nicht immer eine Manifestation der Erkrankung (reduzierte Penetranz). Dass es zu einer HPAH kommt, ist häufiger bei Frauen als bei Männern der Fall [17]. Diese Geschlechterimbalance ist auch von der IPAH und der PAH bei Bindegewebserkrankungen bekannt, die beide eine höhere Prävalenz bei Frauen als bei Männern aufweisen [17]. Wodurch diese Geschlechterunterschiede verursacht werden, ist bisher nicht abschließend geklärt. Eine hereditäre PVOD kommt im Gegensatz zur HPAH und IPAH bei beiden Geschlechtern gleichhäufig vor und hat eine vollständige Penetranz [8]. Die PVOD ist die einzige Form der PAH, die autosomal-rezessiv vererbt wird. Daher müssen biallelische (von beiden Eltern vererbte) Mutationen als homozygote oder compound-heterozygote pathogene Varianten vorliegen. Bisher wurde erst eine Familie beschrieben, bei der es eine autosomal-dominante Komponente einer *EIF2AK4*-Mutation gab, da diese gemeinsam mit einer *BMPR2*-Mutation auftrat und zu einer PAH führte [4].

Bei vereinzelten schweren Fällen von PAH, die bereits im Kindesalter auftraten, wurden homozygote Mutationen in dem Gen *GDF2* beschrieben, dem Gen des BMPR2-Liganden BMP9 [3]. Auch wurden homozygote Mutationen in einem Kalium-Kanal (*KCNK3*) und einem Adenosintriphosphat(ATP)-getriebenen-Kanal (*ATP13A3*; [3]) identifiziert.

## Familienuntersuchungen

Wenn eine genetische Ursache für die Erkrankung identifiziert wurde, ist es wichtig, die Patient*innen darüber aufzuklären, dass auch weitere Familienmitglieder diese Variante tragen bzw. geerbt haben können, jedoch bisher keine Krankheitssymptome aufweisen. Betroffen sein können eigene Kinder, aber auch die Eltern oder Geschwister. Ihnen sollte eine genetische Beratung und prädiktive genetische Diagnostik im Zusammenhang mit einer klinischen Untersuchung angeboten werden. Eine solche wird am Zentrum für Pulmonale Hypertonie unter Leitung von Herrn Prof. Grünig bereits seit mehr als zwei Jahrzehnten angeboten und durchgeführt [9]. Die klinische Untersuchung gesunder Familienmitglieder beinhaltet unter anderem eine körperliche Untersuchung, eine Echokardiographie in Ruhe und unter Belastung, Laboruntersuchungen inklusive des N‑terminalen natriuretischen Propeptids vom B‑Typ (NT-proBNP), eine Lungenfunktionsprüfung und einen 6‑min-Gehtest. Nur in Verdachtsfällen wird eine Rechtsherzkatheteruntersuchung an einem zweiten Termin durchgeführt.

Eine Familienuntersuchung ist wichtig um bei Angehörigen eine PAH so früh wie möglich zu entdecken

Für die genetische Beratung zu einer prädiktiven Testung gesunder Angehöriger ist in Deutschland eine zusätzliche Beratungslizenz erforderlich, insofern die Beratung nicht durch eine Fachärztin bzw. einen Facharzt für Humangenetik erfolgt. In der genetischen Beratung sollten die Vor- und Nachteile einer genetischen Testung erklärt werden. Eine genetische Testung von Kindern ist gegen das Recht der Kinder auf ihr Nichtwissen abzuwägen. Konsequenzen einer vererbten, familiären Mutation für PAH sind ein jährliches nichtinvasives klinisches Screening auf eine PAH sowie eine erhöhte Wachsamkeit beim Auftreten möglicher erster Symptome. Auf diese Weise kann eine Manifestation der PAH so früh wie möglich erkannt und gezielt medikamentös behandelt werden, um Patient*innen so lange wie möglich in der niedrigen Risikoklasse mit einem 1‑Jahres-Mortalitätsrisiko < 5 % zu halten [11]. Eine prädiktive Testung, bei der die familiäre Variante hingegen nicht nachgewiesen werden konnte, reduziert das Risiko, an einer PAH zu erkranken, auf das der Normalbevölkerung.

Eine gezielte, prädiktive genetische Testung richtet sich ausschließlich auf die familiäre Variante. Hierzu wird entweder eine Sanger-Sequenzierung oder eine Methode zum Nachweis von Exondeletionen bzw. -duplikationen („multiplex ligation-dependent probe amplification“ [MLPA]) eingesetzt.

## Von der Genetik zur Therapie

Während alle aktuell zugelassenen Medikamente gegen die PAH primär vasodilatatorisch wirken, wurde aktuell ein neuer Wirkansatz mit dem Medikament Sotatercept in einer Phase-III-Studie untersucht [10]. Es ist ein sogenannter Activin-Liganden-Fänger, der den BMPR2-Signalweg wieder ins Gleichgewicht bringt. Denn nicht nur bei *BMPR2*-Mutationsträgern, sondern auch bei IPAH-Patient*innen ist die *BMPR2*-Expression reduziert [15]. Der BMPR2-Signalweg ist essenziell für die Zellhomöostase, da er Apoptose fördert und Proliferation hemmt. Eine Verringerung von BMPR2 bedeutet daher ein gesteigertes Wachstum der glatten Muskelzellen und Endothelzellen in den Arteriolen. BMPR2 ist Teil des Transforming-growth-factor-β(TGF-β)-Signalwegs. Ein Gegenspieler des BMPR2-Signalwegs ist der Activin-Signalweg, der vermehrt pro-proliferativ wirkt. Sotatercept ist in der Lage, Activin-Liganden abzufangen und die Wirkung des Activin-Signalwegs zu reduzieren. Es ist ein Fusionsprotein aus der Fc-Domäne des Immunglobulins G1 und der extrazellulären Rezeptordomäne des Activin-Rezeptors IIA.

Eine Phase-II-Studie mit Sotatercept bei PAH-Patient*innen mit Funktionsklasse II–III (von IV) nach Weltgesundheitsorganisation (WHO) und stabiler, oft dreifacher Kombinationstherapie konnte nach 24 Wochen in der höchsten Dosierung eindrücklich den pulmonalvaskulären Widerstand um > 3 Wood-Einheiten senken [12]. Die Phase-III-Studie, an der auch die Thoraxklinik Heidelberg beteiligt war, demonstrierte eine Verbesserung des primären Endpunkts, der 6‑min-Gehstrecke, um 41 m im Vergleich zu Placebo nach 24 Wochen [10]. Ebenso verbesserten sich 8 der 9 hierarchisch getesteten sekundären Endpunkte. Aktuell laufen vier weitere Phase-III-Studien. Eine Studie untersucht die Effektivität und Sicherheit von Sotatercept bei japanischen PAH-Patient*innen (ClinicalTrials.gov Identifier NCT05818137), die ZENITH-Studie schließt Patient*innen mit einem 1‑Jahres-Mortalitätsrisiko von > 20 % ein, die HYPERION-Studie rekrutiert Patient*innen mit neu diagnostizierter PAH und einem mittleren oder hohen Mortalitätsrisiko und die SOTERIA-Studie ist als Langzeitnachverfolgungsstudie („long-term follow-up study“) konzipiert. Außerdem wird Sotatercept aktuell in zwei Phase-II-Studien getestet. In der CADENCE-Studie werden Patient*innen mit kombinierter prä- und postkapillärer pulmonaler Hypertonie bei Herzinsuffizienz mit erhaltener Ejektionsfraktion eingeschlossen, in der MOONBEAM-Studie Kinder mit PAH. Neben Sotatercept sind weitere Substanzen in der klinischen Prüfung, die den TGF-β-Signalweg beeinflussen (Tab. 1) oder andere molekulare Ziele wie den Platelet-derived-growth-factor(PDGF)-Rezeptor adressieren.

**Table 1:** 

Substanz	Mechanismus	Studienphase	Endpunkt	Studien-ID (ClinicalTrials.gov Identifier)
PF-07868489	Anti-BMP9: Reduktion des Ligandenlevels	Phase I	Sicherheit und Verträglichkeit	NCT06137742
KER-012	Activin-Rezeptor-IIB-Fusionsprotein als Ligandenfalle für Activine, GDF8 und GDF11	Phase II	PVR-Änderung nach 24 Wochen	NCT05975905
LTP001	SMURF1-Antagonist, Hemmung des BMPR2-Signalweg-Inhibitors	Phase II	Sicherheit, Langzeitnachverfolgung für 52 Wochen	NCT05764265
LTP001	SMURF1-Antagonist, Hemmung des BMPR2-Signalweg-Inhibitors	Phase II	PVR-Änderung nach 25 Wochen	NCT05135000

*BMPR2* „bone morphogenetic protein receptor 2“, *PVR* pulmonalvaskulärer Widerstand, *SMURF1* „SMAD-specific E3 ubiquitin protein ligase 1“, *TGF‑β* „transforming growth factor β“

## Ausblick

Es ist zu erwarten, dass sich der bestehende Therapiealgorithmus der PAH in naher Zukunft um mindestens eine neue Medikamentenklasse erweitern wird. Ziel der Therapien sollte sein, nicht nur die Progression der Erkrankung zu verlangsamen, sondern ein Re-Remodeling, das heißt einen Rückbau der zugewachsenen pulmonal-arteriellen Gefäße zu bewirken. Die Genetik, aber auch weitere molekulare Forschungsfelder wie Analysen des Proteoms, Transkriptoms oder Metaboloms können helfen, die genaue Erkrankungsursache der Patient*innen besser zu verstehen und im Idealfall eine maßgeschneiderte Therapie anzubieten.

## Fazit für die Praxis

- Eine genetische Testung ist sinnvoll für Patient*innen mit idiopathischer pulmonal-arterieller Hypertonie (PAH), hereditärer PAH, pulmonaler venookklusiver Erkrankung, medikamenten- oder toxininduzierter PAH, PAH bei angeborenen Herzfehlern oder PAH bei Kindern sowie bei Patient*innen, bei denen der Verdacht auf eine familiäre Häufung besteht.
- Eine positive genetische Testung kann Differenzialdiagnosen sichern und ermöglicht die prädiktive Testung von Familienangehörigen, um bei ihnen eine PAH so früh wie möglich zu erkennen.
- Eine solche Testung sollte, wie in der Thoraxklinik Heidelberg und dem Institut für Humangenetik der Universität Heidelberg angeboten, über eine Genpanelsequenzierung in einem zertifizierten Labor durchgeführt werden, welches das Panel fortlaufend aktualisiert und an den aktuellen Stand des Wissens anpasst.
